# International circumpolar surveillance: update on the interlaboratory quality control program for *Streptococcus pneumoniae*, 2009 to 2020

**DOI:** 10.1128/spectrum.04245-23

**Published:** 2024-04-23

**Authors:** Alyssa R. Golden, Averil Griffith, Brenna C. Simons, Alisa Reasonover, Hans-Christian Slotved, Brigitte Lefebvre, Karl G. Kristinsson, Donna Hurteau, Gregory J. Tyrrell, Michael G. Bruce, Irene Martin

**Affiliations:** 1 National Microbiology Laboratory, Public Health Agency of Canada, Winnipeg, Manitoba, Canada; 2 Arctic Investigations Program, Centers for Disease Control and Prevention, Anchorage, Alaska, USA; 3 Neisseria and Streptococcus Reference Laboratory, Department of Bacteria, Parasites and Fungi, Statens Serum Institut, Copenhagen, Denmark; 4 Laboratoire de santé publique du Québec, Sainte-Anne-de-Bellevue, Québec, Canada; 5 Department of Clinical Microbiology, Landspitali – the National University Hospital of Iceland, Reykjavik, Iceland; 6 Alberta Precision Laboratory – Public Health Laboratory and Division of Diagnostic and Applied Microbiology, Department of Laboratory Medicine and Pathology, University of Alberta, Edmonton, Alberta, Canada; Yale School of Public Health, New Haven, Connecticut, USA

**Keywords:** *Streptococcus pneumoniae*, quality control, serotyping, antimicrobial susceptibility, circumpolar surveillance, invasive bacterial disease

## Abstract

The International Circumpolar Surveillance (ICS) program is a population-based surveillance network for invasive bacterial diseases throughout Arctic countries and territories. The ICS quality control program for *Streptococcus pneumoniae* serotyping and antimicrobial susceptibility testing has been ongoing since 1999. Current participating laboratories include the Provincial Laboratory for Public Health in Edmonton, Alberta; Laboratoire de santé publique du Québec in Sainte-Anne-de-Bellevue, Québec; the Centers for Disease Control’s Arctic Investigations Program in Anchorage, Alaska; the Neisseria and Streptococcus Reference Laboratory at Statens Serum Institut in Copenhagen, Denmark; the Department of Clinical Microbiology, Landspitali in Reykjavik, Iceland; and Public Health Agency of Canada’s National Microbiology Laboratory in Winnipeg, Manitoba. From 2009 to 2020, 140 isolates of *S. pneumoniae* were distributed among the six laboratories as part of the quality control program. Overall serotype concordance was 96.9%, with 99.3% concordance to pool level. All participating laboratories had individual concordance rates >92% for serotype and >97% for pool. Overall concordance by modal minimum inhibitory concentration (MIC) for testing done by broth microdilution or Etest was 99.1%, and >98% for all antimicrobials tested. Categorical concordance was >98% by both CLSI and EUCAST criteria. For two laboratories performing disc diffusion, rates of concordance by modal MIC were >97% for most antimicrobials, except chloramphenicol (>93%) and trimethoprim/sulfamethoxazole (>88%). Data collected from 12 years of the ICS quality control program for *S. pneumoniae* demonstrate excellent (≥95%) overall concordance for serotype and antimicrobial susceptibility testing results across six laboratories.

**IMPORTANCE:**

Arctic populations experience several social and physical challenges that lead to the increased spread and incidence of invasive diseases. The International Circumpolar Surveillance (ICS) program was developed to monitor five invasive bacterial diseases in Arctic countries and territories. Each ICS organism has a corresponding interlaboratory quality control (QC) program for laboratory-based typing, to ensure the technical precision and accuracy of reference testing services for these regions, and identify and correct potential problems. Here, we describe the results of the ICS *Streptococcus pneumoniae* QC program, from 2009 to 2020. Excellent overall concordance was achieved for serotype and antimicrobial susceptibility testing results across six laboratories. Ongoing participation in these QC programs ensures the continuation of quality surveillance systems within Arctic populations that experience health disparities.

## INTRODUCTION

Arctic populations face a series of unique challenges, both social and physical, that lead to the increased spread and incidence of invasive diseases. The harsh northern climate forces residents indoors; however, small isolated communities often have inadequate housing; crowded households and poor ventilation can lead to increased person-to-person transmission of infectious diseases (1, 2). Improper or overuse of antimicrobial agents in some remote communities with poorly developed or poorly supported public health systems has led to, and may continue to support, the emergence of resistant bacterial clones (1). There are also significant health inequities between Indigenous and non-Indigenous people living in circumpolar regions, where Indigenous populations tend to fare worse for a large number of health indicators (3, 4).

To improve surveillance, prevention, and disease control in Arctic populations experiencing health disparities, the International Circumpolar Surveillance (ICS) program was implemented in 1999, to monitor invasive pneumococcal disease (IPD) in Alaska and across the Canadian Arctic (2). Initially, participating laboratories for IPD surveillance included the National Centre for Streptococcus at the Provincial Laboratory for Public Health (PLPH) in Edmonton, Alberta; Laboratoire de santé publique du Québec (LSPQ) in Sainte-Anne-de-Bellevue, Québec; and the Centers for Disease Control’s Arctic Investigations Program (AIP) in Anchorage, Alaska. This collaboration provided an opportunity to expand Canada’s previously existing Interlaboratory Quality Control (QC) program for *Streptococcus pneumoniae* to include AIP.

In August 2004, the ICS QC program for *S. pneumoniae* was expanded to include the Neisseria and Streptococcus Reference Laboratory (previously the WHO Collaborating Centre for Reference and Research on Pneumococci) at Statens Serum Institut (SSI) in Copenhagen, Denmark. In September 2006, the Department of Clinical Microbiology at Landspitali—the National University Hospital of Iceland in Reykjavik joined the ICS program. On 1 April 2010, national streptococcus laboratory services in Canada were transferred from PLPH to the National Microbiology Laboratory (NML) in Winnipeg, Manitoba, and NML was added to the interlaboratory QC program bringing the number of participating laboratories to six.

The purpose of the QC program is to provide an external proficiency testing mechanism for pneumococcal serotyping and antibiotic susceptibility testing. The *S. pneumoniae* QC panel serves to identify and correct potential problems in reagents and equipment, test interpretation, and assess technical precision and accuracy. A previous publication by Reasonover et al. detailed the first 10 years of the *S. pneumoniae* QC program, from 1999 to 2008 (5). The current report describes the results of the *S. pneumoniae* ICS Interlaboratory QC program collected from 2009 to 2020.

## MATERIALS AND METHODS

### Distribution of isolates

From 2009 through 2016, five of the six participating laboratories (AIP, LSPQ, NML, PLPH, and SSI) were responsible for distributing one set of seven *S*. *pneumoniae* isolates every 30 months (overall, two distributions per year). Due to time and international shipping constraints, the distribution schedule was changed in 2017 to one distribution per year, with participating laboratories responsible for sending out one panel every 5 years. The distribution dates and laboratory schedule were agreed upon in advance. Seven phenotypically characterized *S. pneumoniae* isolates were selected by the distributing laboratory to represent a variety of serotypes and antimicrobial resistance patterns. All isolates were transported using charcoal transport media or chocolate agar slants and shipped according to International Air Transportation Association regulations. Participants cultured isolates according to Clinical and Laboratory Standards Institute (CLSI)/European Committee on Antimicrobial Susceptibility Testing (EUCAST) guidelines (6, 7).

From this point forward in the manuscript, to maintain confidentiality, participating laboratories will be referred to as laboratories A through F.

### Serotyping

Serotyping was performed by Quellung reaction using commercial antisera (SSI Diagnostica, Hillerød, Denmark) (8), and according to the routine testing methodology in use at each laboratory. Laboratories A, B, C, and E maintain a complete collection of antisera which enables them to classify the serotypes for which there are commercial antisera available. Laboratories D and F maintain antisera that permit grouping and factoring for the most common serogroups including 3, 6, 9, 19, and 23. A serotyping result was considered correct if it was consistent with the results identified by the submitting laboratory using the antisera available in each laboratory. Serotype discrepancies between laboratories were grouped into one of two categories: cross-reactions of common factors or those with no explanation. Cross-reactions of common factors occurred when the isolate reacted with antibodies shared by more than one serogroup or serotype. Unexplained discrepancies included the following: reporting non-typeable for an isolate with a confirmed serotype; reporting a different serotype within a serogroup (factoring) or different serogroup/type within a pool; and incomplete factoring when a complete antisera collection was available.

### Antimicrobial susceptibility testing

Antimicrobial compounds of interest in this study included the following: penicillin, ceftriaxone, cefotaxime, chloramphenicol, clindamycin, erythromycin, levofloxacin, trimethoprim/sulfamethoxazole, and vancomycin. For each participating laboratory, minimum inhibitory concentrations (MICs) were determined for antimicrobials routinely tested by that laboratory, according to their routine testing method. Table 1 describes the antimicrobials tested by each participating laboratory for each panel. Laboratories A, B, C, and E reported MIC results based on the TREK Sensititre broth microdilution method. Exceptions to this included the following: 2009 where laboratory B reported disc diffusion zone diameters for chloramphenicol and trimethoprim/sulfamethoxazole, and Etest results for other antimicrobials; and 2018 onwards, where laboratory A reported results from Vitek AST-ST03 cards. Laboratory F used reference broth microdilution and panels prepared in-house according to CLSI guidelines (9). Finally, laboratory D reported Etest results for penicillin and ceftriaxone, and disc diffusion zone diameters for the remaining antimicrobials. MIC results were expected to be within one log_2_ dilution of the modal MIC, regardless of MIC testing methodology. The modal MIC represents the most frequently reported MIC value for a given isolate-antimicrobial combination (assumed to be the value that is closest to the true MIC). When two MIC values were reported with equal frequency, both were accepted as a modal value; if there was no modal MIC, the isolate was removed from further calculations. Cefotaxime results from the 2009A and 2009B panels were not included in this report, as only two participating laboratories tested that drug in 2009.

**TABLE 1:** Antimicrobials of the standard set of nine that were not tested by each of six participating ICS laboratories for each panel^*f*^

ICS QC site	Test method	Panel distribution year; antimicrobials that were NOT tested^*a*^																			
		2009A	2009B	2010A	2010B	2011A	2011B	2012A	2012B	2013A	2013B	2014A	2014B	2015A	2015B	2016A	2016B	2017	2018	2019	2020
A	BMD	✓	✓	✓	✓	✓	✓	✓	✓	✓	✓	✓	✓	✓	✓	✓	✓	✓	✓	✓	✓
B^*b*^	BMD (E-test/disc diffusion in 2009 only)	CTX, LEV, VAN	CTX, LEV, VAN	✓	✓	✓	✓	✓	✓	✓	✓	✓	✓	✓	✓	✓	✓	✓	✓	✓	✓
C	BMD	DNP	DNP	DNP	✓	✓	✓	✓	✓	✓	✓	✓	✓	✓	✓	✓	✓	✓	✓	✓	✓
D^*c*^	E-test/disc diffusion	CTX	CTX	VAN, CTX	CTX	CTX	CTX	CTX	CTX	CTX	CTX	CTX	CTX	CTX	CTX	CTX	CTX	CTX	CTX	CTX	CTX
E^*d*^	BMD	CRO	CRO	CRO	CRO	CRO	✓	✓	✓	✓	✓	✓	✓	✓	✓	✓	✓	✓	CTX	CTX	✓
F^*e*^	BMD	CTX	CTX	CTX	CTX	CTX	CTX	CTX	CTX	CTX	CTX	CTX	CTX	CTX	CTX	CTX, CHL	CTX, CHL	CTX, CHL	CTX, CHL	CTX, CHL	CTX,CHL

^*a*^ The full set of antimicrobials included the following: penicillin (PEN), ceftriaxone (CRO), cefotaxime (CTX), chloramphenicol (CHL), clindamycin, erythromycin, levofloxacin (LEV), trimethoprim/sulfamethoxazole and vancomycin (VAN). A check mark indicates that all nine antimicrobials were tested for that panel distribution.

^*b*^ CTX, LEV, and VAN were not regularly tested by this laboratory prior to 2010. For 2009, CHL and SXT were tested by disc diffusion, and E-test for other antimicrobials.

^*c*^ CTX is not regularly tested by this laboratory. VAN was not tested for the 2010A panel. PEN and CRO tested by Etest, all others by disc diffusion.

^*d*^ CRO was not regularly tested at this laboratory prior to the 2011B panel. CTX was not tested for the 2018 and 2019 panels due to a brief change in panel composition.

^*e*^ CTX is not regularly tested by this laboratory. Beginning in 2016, CHL was no longer regularly tested.

^*f*^ BMD, broth microdilution; DNP, laboratory did not participate in this panel distribution.

Interpretive categorical concordance [susceptible, intermediate (if appropriate), resistant] was also reported for all MIC and disc results, using both CLSI (Performance Standards for Antimicrobial Susceptibility Testing; Informational Supplement; M100 series) (6) and EUCAST criteria (7). MICs and zone diameters were evaluated for correlation with the expected interpretive category consistent with the modal MIC. For CLSI interpretations, categorical agreement for penicillin and ceftriaxone/cefotaxime used the CLSI M100 oral penicillin V and non-meningitis breakpoints, respectively (6). For EUCAST interpretations, the categorical agreement used the benzylpenicillin, ceftriaxone, and cefotaxime breakpoints for indications other than meningitis (7). Categorical interpretive errors were classified as follows: minor (intermediate to susceptible/resistant OR susceptible/resistant to intermediate), major (false resistance), or very major (false susceptibility). MIC results for *S. pneumoniae* ATCC 49619 were reported along with each QC panel and were expected to be within the range of the currently published CLSI M100 standards.

Exact MIC values for some antimicrobials could not be determined due to the differing MIC endpoints used in each laboratory. If different MIC endpoint ranges were reported, the lowest “>” MIC value or the highest “<” MIC value was used as the modal MIC if the reported MIC values were within the expected range. When an Etest MIC did not correspond to the traditional doubling dilution MIC, the MIC was rounded up to the next highest log_2_ dilution to compare with the other participating laboratories.

### Reporting

A standardized report form was provided by the distributing laboratory for each QC panel. Participating laboratories were expected to include their test methods, serotypes, and MIC results on this form and return to the distributing laboratory within 6 weeks of panel receipt. The distributing laboratory was responsible for compiling all results into a summary report for distribution among the other laboratories. The summary report identified and discussed any discrepant results, as well as noted any pertinent points for follow-up, discussion, and informal troubleshooting with the group.

In this manuscript, percent concordance is referred to as excellent (≥95% concordance), very good (>90% to <95%), good (>85 to ≤90%), or satisfactory (80 to ≤85%) (10).

### Ownership of isolates

QC isolates are the property of the province, state, or country from which they were distributed. Isolates may have been retained by participating laboratories for internal reference use; however, the use for research purposes or further distribution to external laboratories was not to occur without the written consent of the distributing laboratory.

This activity was reviewed by the CDC and was conducted consistent with applicable federal law and CDC policy. See for example, 45 C.F.R. part 46, 21 C.F.R. part 56; 42 U.S.C.§241(d); 5 U.S.C. §552a; 44 U.S.C. §3501 et seq.

## RESULTS

### Serotyping

**Fig 1:**
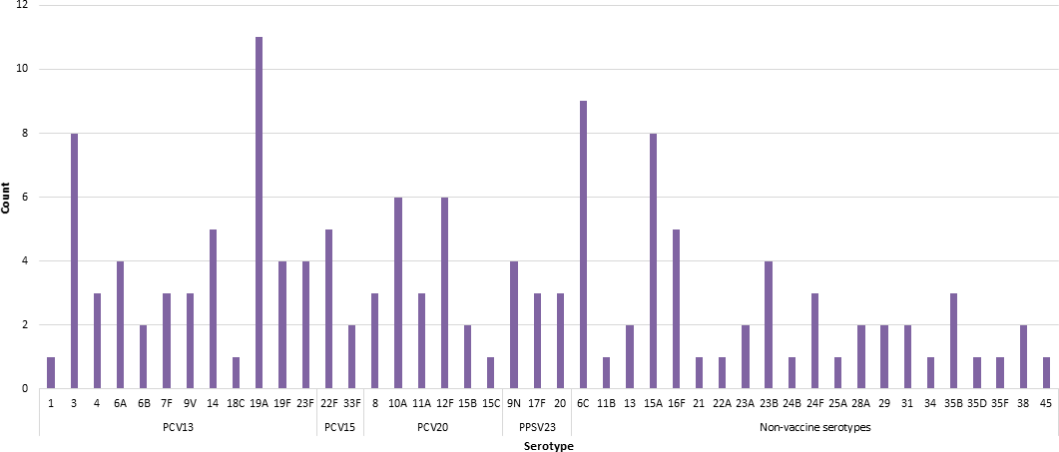
Serotypes represented in the ICS *S. pneumoniae* QC panel, 2009–2020.

From 2009 through 2020, a total of 140 isolates representing 44 serotypes were distributed among the participating laboratories (Fig. 1). The most common serotypes were 19A, 6C, 15A, 3, and 10A, representing 30% of isolates distributed. For the 140 isolates distributed across six laboratories, there were 811 comparisons made for the calculation of concordance. In accordance with the antisera available at each laboratory, serotype concordance was 96.9% (786/811), with 99.3% (805/811) concordance to the pool level. All participating laboratories had individual concordance rates >92% for serotype and >97% for pool (Table 2). Discrepancies between the expected serotype and reported results were noted in 19 instances (Table 3). In all, 16 discrepancies were categorized as unexplained, including one reported as non-typeable by three participating laboratories when the isolate had a confirmed serotype (6B) and four reported as a different serotype within a serogroup (three of which involved serogroup 6). Five additional isolates were reported as a different serogroup/type within a pool, including two each from Pools D and H, and one from Pool C. One isolate was reported as a serotype from a different pool, four had incomplete factoring when a complete antisera collection was available (all serogroup 24), and one had disparate results from two separate participating laboratories; one reported a different serogroup/type within a pool and the second reported a serotype from a different pool. Cross-reaction of common factors occurred for the remaining three discrepancies, specifically for serotypes 35B, 35D, and 29 (Pool G).

**TABLE 2:** Comparison of serotyping results and concordance for 140 *S*. *pneumoniae* from six ICS laboratories

ICS QC laboratory	Number of isolates tested^*a*^	Concordance by antisera available at each laboratory^*b*^		Concordance by pool	
		Number of correct results	Percent concordance	Number of correct results	Percent concordance
A	140	138	98.6	139	99.3
B	140	136	97.1	139	99.3
C	119	119	100	119	100
D	134	124	92.5	131	97.8
E	140	133	95.0	140	100
F	138	136	98.6	137	99.3
**Total**	811	786	**96.9**	805	**99.3**

^*a*^ Number of tested isolates varied due to non-viable isolates following shipping, and laboratory C not participating until the 2010B distribution.

^*b*^ Participants D and F maintain antisera that permit grouping and factoring for the most common serogroups.

**TABLE 3:** Discordant *S. pneumoniae* serotype results reported by each participating ICS laboratory, 2009 to 2020

Isolate ID	Serotype result by laboratory^*a*^							Discrepancy type
	A		B	C	D^*b*^	E	F^*b*^	
09A-03	29		35B	DNP	15	35B	35, 29, or 42	Shared factor
09A-05	6C		6C	DNP	6B	6C	6	Unexplained
10B-09	NT		NT	6B	6B	6B	NT	Unexplained
11A-07	6C		6C	6C	6A	6C	6C	Unexplained
12A-01	11A		11A	11A	36	11A	11A	Unexplained
12B-11	24F		24F	24F	Pool C	24^*c*^	Pool C	Unexplained
13A-02	16F		11A	16F	37	16F	16	Unexplained
14A-02	17F		22A	17F	10	17F	17	Unexplained
14B-09	6B		6B	6B	6B	6C	6B	Unexplained
15A-02	24F		24F	24F	Pool C	24^*c*^	Pool C	Unexplained
15A-07	24B		24B	24B	Pool C	24^*c*^	NT	Unexplained
15B-12	20		20	20	24, 21 or 40	20	20	Unexplained
16A-01	23B		23B	23B	23F	23B	23B	Unexplained
16A-02	24F		24F	24F	NG	24^*c*^	NG	Unexplained
16B-10	14		14	14	23B	14	Pool H	Unexplained
2018-01	29		29	29	29	35B	Pool G	Shared factor
2018-04	28A		28A	28A	Pool H	28A	23	Unexplained
2018-07	29		35D	35D	Pool G	35B	Pool G	Shared factor
2020-04	23B		23B	23B	6C	23B	23B	Unexplained

^*a*^ DNP, did not participate in this panel distribution; NT, nontypeable; NG, no growth. The correct serotype is underlined in the column of the submitting laboratory.

^*b*^ Participants D and F maintain antisera that permit grouping and factoring for the most common serogroups.

^*c*^ Participant E maintains a full set of typing antisera but only reported to the group level.

### Antimicrobial susceptibility testing

For 140 *S*. *pneumoniae* isolates tested by broth microdilution, Vitek, or Etest, there was a total of 6,004 MIC results available for comparison, ranging between 483 (cefotaxime) and 811 (penicillin) comparisons depending on the antimicrobial (Table 4). Seven isolates had variable results between participating laboratories and therefore did not have a modal MIC; antimicrobials affected included chloramphenicol (*n* = 2), clindamycin (*n* = 1), erythromycin (*n* = 3), and trimethoprim/sulfamethoxazole (*n* = 1). There was no explanation for the variability in test results in all cases except for the clindamycin isolate; the isolate in question possessed the inducible clindamycin resistance phenotype, which is rare for *S. pneumoniae*. As the inducible phenotype cannot be detected by broth microdilution, four participating laboratories did not report the correct results. Aside from the distributor, only the participant performing disc diffusion/D-test was able to identify the phenotype.

**TABLE 4:** Comparison of MIC results and concordance of modal MIC^*a*^ and categorical interpretations for 140 *S*. *pneumoniae* from six ICS laboratories using broth microdilution or Etest

Antimicrobial	No. of pairwise comparisons with MICs that differed from the modal MIC^*a*^ (log_2_ dilutions) by:							Total no. pairwise comparisons	% Concordance by:		
									Modal MIC (±1 log_2_)	Categorical interpretation of modal MIC	
	≤ –3	–2	–1	Same	+1	+2	≥+3			CLSI^*b*^	EUCAST^*c*^
Penicillin	2	8	56	688	53	3	1	811	98.3 (797/811)	96.3 (781/811)	96.3 (781/811)
Ceftriaxone	0	6	44	711	12	2	1	776	98.8 (767/776)	98.5 (764/776)	97.8 (759/776)
Cefotaxime	0	1	15	455	12	0	0	483	99.8 (482/483)	98.6 (476/483)	99.4 (480/483)
Chloramphenicol	0	2	74	495	41	1	0	613	99.5 (610/613)	97.7 (599/613)	99.2 (608/613)
Clindamycin	0	2	4	631	26	6	3	672	98.4 (661/672)	99.0 (665/672)	99.3 (667/672)
Erythromycin	1	2	18	625	12	3	3	664	98.6 (655/664)	99.1 (658/664)	99.4 (660/664)
Levofloxacin	0	0	48	591	22	0	1	662	99.8 (661/662)	99.8 (661/662)	100 (662/662)
Trimethoprim/sulfamethoxazole	0	2	23	595	36	2	2	660	99.1 (654/660)	97.3 (642/660)	96.2 (635/660)
Vancomycin	0	0	2	653	8	0	0	663	100 (663/663)	100 (663/663)	100 (663/663)
**Overall**	**2**	**17**	**246**	**5,142**	**185**	**9**	**8**	**6,004**	**99.1** (5,950/6,004)	**98.4** (5,909/6,004)	**98.5** (5,915/6,004)

^*a*^ For this report, isolates with no modal MIC were excluded from calculations; cefotaxime results from 2009 were not included as there were only two participant laboratories reporting results.

^*b*^ Using CLSI M100, 33rd Edition, 2023. For β-lactams, breakpoints used were oral penicillin V, ceftriaxone, and cefotaxime non-meningitis.

^*c*^ Using EUCAST Breakpoint tables for interpretation of MICs, Version 13.0, 2023. For β-lactams, breakpoints used were benzylpenicillin; ceftriaxone, and cefotaxime for indications other than meningitis.

Overall concordance by modal MIC was 99.1%, and >98% for all compounds tested. The categorical interpretation was also excellent, with 98.4% and 98.5% overall concordance using CLSI and EUCAST interpretive criteria, respectively. A comparison of the modal MIC interpretation to disc diffusion results submitted by two participating laboratories is presented separately (Table 5). For these laboratories, rates of concordance in comparison to the modal MIC were excellent (>97%) for clindamycin, erythromycin, levofloxacin, and vancomycin. For chloramphenicol, concordance was very good (93.8%) using CLSI breakpoints and excellent (95.2%) by EUCAST. For trimethoprim/sulfamethoxazole, rates were comparatively lower at 88.4% (good) and 91.1% (very good) using CLSI and EUCAST breakpoints, respectively.

**TABLE 5:** Comparison of disc diffusion and modal MIC^*a*^ categorical interpretations for 140 *S*. *pneumoniae* results from two ICS laboratories using disc diffusion for six antimicrobials^*a*^

Antimicrobial	Number of isolates tested^*a*^^,^^*b*^^,*c*^	% Concordance of categorical interpretation of modal MIC	
		CLSI^*d*^	EUCAST^*e*^
Chloramphenicol	145	93.8 (136/145)	95.2 (138/145)
Clindamycin	133	97.7 (130/133)	98.5 (131/133)
Erythromycin	131	99.2 (131/132)	99.2 (131/132)
Levofloxacin	134	100 (120/120)	100 (120/120)
Trimethoprim/sulfamethoxazole	146	88.4 (129/146)	91.1 (133/146)
Vancomycin	127	100 (127/127)	100 (127/127)
Total	816	94.7 (773/816)	95.6 (780/816)

^*a*^ One participating laboratory tested all six listed compounds by disc diffusion consistently for all panels; one participant performed disc diffusion for the 2009AB panels for chloramphenicol and trimethoprim/sulfamethoxazole only.

^*b*^ Number of tested isolates varied due to non-viable isolates following shipping, and vancomycin not being tested in the first distribution.

^*c*^ For this report, isolates with no modal MIC were excluded from calculations.

^*d*^ Using CLSI M100, 33rd Edition, 2023.

^*e*^ Using EUCAST Breakpoint tables for interpretation of MICs and zone diameters, Version 13.0, 2023.

Agreement by modal MIC for the β-lactam antimicrobials was excellent, with 98.3%, 98.8%, and 99.8% concordance for penicillin, ceftriaxone, and cefotaxime, respectively (Table 4). Categorical agreement for penicillin was 96.3% (29 minor errors, 1 very major error) and 96.3% (30 minor errors) for CLSI and EUCAST interpretive criteria, respectively (Tables 4 and 6). The majority of minor errors resulted from a difference in one doubling dilution: 0.06 μg/mL to 0.12 μg/mL (the difference between susceptible and intermediate for both sets of criteria); 1 μg/mL to 2 μg/mL (the difference between intermediate and resistant for CLSI criteria); or 2 μg/mL to 4 μg/mL (the difference between intermediate and resistant for EUCAST criteria). The very major error by CLSI breakpoints was a large, unexplained difference of five doubling dilutions (0.06 → 2 µg/mL). Concordance by CLSI and EUCAST criteria was also excellent for ceftriaxone [98.5% (12 minor errors), 97.8% (17 minor errors)] and cefotaxime [98.6% (7 minor errors), 99.4% (3 minor errors)]. The majority of minor errors resulted from a difference in one doubling dilution on either side of the breakpoint for susceptible and intermediate, for both sets of interpretive criteria.

**TABLE 6:** Interpretive categorical agreement based on modal MIC category for 140 *S*. *pneumoniae* from six ICS laboratories using broth microdilution, Etest, or disc diffusion^*c*^

ICS QC laboratory	# of results available	CLSI^*a*^		EUCAST^*b*^	
		Categorical errors	Categorical agreement (%)	Categorical errors	Categorical agreement (%)
A	1,239 MIC	13 MI (4 PEN, 1 CRO, 4 CTX, 4 SXT); 2 VM (CHL)	98.8 (1,224/1,239)	7 MI (4 PEN, 1 CRO, 2 SXT); 1 MA (SXT)	99.4 (1,231/1,239)
B	1,185 MIC; 26 DD	12 MI (6 PEN, 1 CLI, 5 SXT); 17 MA (14 CHL, 1 CLI, 1 ERY, 1 SXT); 6 VM (1 CHL, 1 ERY, 4 SXT)	97.1 (1,176/1,211)	18 MI (5 PEN, 4 CRO, 2 CTX, 7 SXT); 10 MA (8 CHL, 1 CLI, 1 ERY); 6 VM (1 ERY, 5 SXT)	97.2 (1,177/1,211)
C	1,067 MIC	12 MI (2 PEN, 3 CRO, 3 CTX, 4 SXT); 1 VM (CHL)	98.8 (1,054/1,067)	7 MI (4 PEN, 1 CRO, 1 CTX, 1 SXT); 1 VM (SXT)	99.3 (1,059/1,067)
D	268 MIC; 791 DD	32 MI (11 PEN, 6 CRO, 2 CLI, 1 ERY, 12 SXT); 1 MA (SXT); 3 VM (CHL)	96.6 (1,023/1,059)	23 MI (8 PEN, 8 CRO, 7 SXT); 3 MA (2 CLI, 1 SXT); 2 VM (1 ERY, 1 SXT)	97.4 (1,031/1,059)
E	1190 MIC	7 MI (1 PEN, 2 CLI, 2 ERY, 2 SXT); 3 MA (2 CHL, 1 LEV), 1 VM (PEN)	99.1 (1,179/1,190)	7 MI (3 PEN, 4 SXT); 2 MA (1 CLI, 1 ERY); 1 VM (SXT)	99.2 (1,180/1,190)
F	1,055 MIC	12 MI (5 PEN, 2 CRO, 1 CLI, 2 ERY, 2 SXT); 2 MA (1 CHL, 1 CLI)	98.7 (1,041/1,055)	11 MI (6 PEN, 3 CRO, 2 SXT); 3 MA (2 CLI, 1 ERY); 1 VM (CHL)	98.6 (1,040/1,055)
**Total**	6,004 MIC, 817 DD	88 MI (29 PEN, 12 CRO, 7 CTX, 6 CLI, 5 ERY, 29 SXT); 23 MA (17 CHL, 2 CLI, 1 ERY, 1 LEV, 2 SXT); 13 VM (1 PEN, 7 CHL, 1 ERY, 4 SXT)	**98.2** (6,697/6,821)	73 MI (30 PEN, 17 CRO, 3 CTX, 23 SXT); 19 MA (8 CHL, 6 CLI, 3 ERY, 2 SXT); 11 VM (1 CHL, 2 ERY, 8 SXT)	**98.5** (6,718/6,821)

^*a*^ Using CLSI M100, 33rd Edition, 2023. For β-lactams, breakpoints used were as follows: oral penicillin V, ceftriaxone, and cefotaxime non-meningitis.

^*b*^ Using EUCAST Breakpoint tables for interpretation of MICs, Version 13.0, 2023. For β-lactams, breakpoints used were benzylpenicillin; ceftriaxone, and cefotaxime for indications other than meningitis.

^*c*^ MI, minor error (intermediate to susceptible/resistant OR susceptible/resistant to intermediate); MA, major error (false resistance); VM, very major error (false susceptibility); PEN, penicillin; CRO, ceftriaxone; CTX, cefotaxime; CHL, chloramphenicol; CLI, clindamycin; ERY, erythromycin; LEV, levofloxacin; SXT, trimethoprim/sulfamethoxazole.

Concordance by modal MIC, CLSI, and EUCAST breakpoints for chloramphenicol was 99.5%, 97.7%, and 99.2%, respectively. By EUCAST criteria, there were eight major errors and one very major error in comparison to the modal MIC interpretation. Using CLSI criteria, there were 17 major and 7 very major errors (including three by disc diffusion); this accounted for over half of the very major errors (7/13) noted using CLSI criteria. Neither set of interpretive criteria incorporates an intermediate category for chloramphenicol, which may have contributed to the high number of very major errors for this compound.

Concordance for clindamycin and erythromycin was >98% across modal MIC, CLSI, and EUCAST breakpoints (Table 4). For clindamycin, there were eight (six minor and two major) and six (all major) errors by CLSI and EUCAST breakpoints, respectively (including two for each by disc diffusion). Many of these major errors were large, unexplained differences that were considered errors across all three metrics. For erythromycin, there were seven (five minor, one major, one very major) errors by CLSI, including one minor error by disc diffusion. By EUCAST, there were five (three major and two very major) errors, with one of the very major errors being by disc diffusion. The other very major error by EUCAST (the same very major error by CLSI) was a large, unexplained difference of at least four doubling dilutions (≤0.12 → >2 µg/mL).

For trimethoprim/sulfamethoxazole, concordance was 99.1% for modal MIC, but 97.3% and 96.2% of categorical agreement for CLSI and EUCAST breakpoints, respectively. The most common type of error was minor, with 35 (29 minor, 2 major, and 4 very major) and 26 (23 minor, 2 major, and 8 very major) errors by CLSI and EUCAST breakpoints, respectively. The majority of minor errors resulted from a difference in one doubling dilution on either side of the breakpoint for susceptible and intermediate, for both sets of interpretive criteria. Trimethoprim/sulfamethoxazole accounted for a majority of the very major errors (8/11) noted using EUCAST criteria; five very major errors were by disc diffusion, and the others were a difference of 2 doubling dilutions across the breakpoints.

Concordance for levofloxacin was high at 99.8% by modal MIC and CLSI breakpoints (including one major error), and 100% by EUCAST breakpoints. There was 100% concordance by modal MIC and no categorical errors for vancomycin.

## DISCUSSION

The ICS QC program for *S. pneumoniae* is critical to ensure that accurate testing results are being provided to clinical and laboratory partners in vulnerable Arctic communities. Our QC data demonstrate excellent concordance for serotype and antimicrobial susceptibilities for 140 *S*. *pneumoniae* isolates distributed across six laboratories in North America and Europe. Considering the antisera available at each laboratory, serotype concordance was 96.9%, with 99.3% concordance to pool level. For antimicrobial susceptibility testing, overall concordance by modal MIC was 99.4% (>98% for all compounds tested) and overall categorical concordance using CLSI and EUCAST interpretive criteria was 98.4% and 98.5%, respectively. For two participating laboratories performing a portion of testing by disc diffusion, rates of concordance in comparison to the modal MIC were >97% for most antimicrobials.

New, higher-valency pneumococcal conjugate vaccines (15-valent, 20-valent formulations), which would replace existing 10- and 13-valent formulations, have been approved for use around the world, including in Canada, United States, and European Union member states (11–13). During this transitionary time, it will be increasingly important to monitor regional serotype distributions, assess vaccine coverage, and track the emergence of non-vaccine types. External quality assurance (EQA) programs will remain a crucial tool to assess the quality of pneumococcal serotyping provided by reference laboratories. An early EQA for pneumococcal serotyping was developed for the Sistema Regional de Vacunas (SIREVA) project in Central and South America, as described by Lovgren et al. (14). Overall serotyping accuracy for phase II of the EQA program development (1999–2005) was 93.8% for 130 isolates (23 vaccine-related and 26 non-vaccine-related) over 13 distributions to 20 participating countries (14). In a more recent study, Slotved et al. describe an EQA scheme performed by eight reference laboratories in countries across Europe and the Middle East, consisting of 22 distributions spread across 11 years (2005–2016) (15). Of 154 isolates (representing 49 serotypes), discrepant results for one or more participants were only identified for 7.1% of isolates. Their results showed that comparable serotyping results can be obtained across various typing methods, including latex agglutination, Quellung reaction, whole-genome sequencing, and various PCR methodologies (15). Our results compare favorably to those from these two manuscripts, as well as to the results of the previous decade of ICS QC results, of 95.8% and 97.4% concordance by serotype and serogroup, respectively (5). Lastly, the World Health Organization’s Global Invasive Bacterial Vaccine Preventable Disease (IB-VPD) Surveillance Network (GISN) provides a yearly EQA panel consisting of *Haemophilus influenzae*, *Neisseria meningitidis*, and *S. pneumoniae*. Litt et al. describe the results from the 2014–2019 exercise, where each panel contained 2–3 *S. pneumoniae* for identification and serotyping per year; the serotyping exercise was mandatory for regional reference laboratories (RRLs) and optional for sentinel site laboratories and national laboratories (SSLs/NLs), depending on resources and capability (16). GISN’s nine RRLs had high serotype concordance, with only a single error across six distributions. SSLs/NLs had low participation (16%–33%), and the serotyping success rate varied between 33% and 100%, depending on the serotype. However, there was a significant upward trend over time in the number of correct serotyping results for these laboratories, as resources and training improved during the reported 6-year time span (16).

One aspect noted by Slotved et al.’s and Litt et al.’s studies was difficulty with concordance when the expected serotype had known cross-reaction of common factors, specifically for serotypes 35B and 29 (15, 16). Our report describes two similar discrepancies, where some participating laboratories identified a serotype 29 isolate as serotype 35B. A second, related discrepancy in our report surrounded the recently described novel serotype 35D (17). Serotype 35D differs from 35B due to the presence of inactivating mutations in *wciG*; as 35D does not react with group 35 antiserum, the type may be missed if all relevant antisera are not used for screening. Serotype 35D can also easily be mistaken for serotype 29, as it differs in serological profile by only one factor serum (35b). It is crucial to serotype Pool G pneumococci with caution, due to the numerous antiserum cross-reactions that may lead to incorrect results. If an isolate tests positive by Pool G antisera, all pertinent group, type and factor sera should be tested each time.

There are few other reports describing external proficiency results for antimicrobial susceptibility testing of *S. pneumoniae*. The ICS QC report for the 1999 to 2008 *S*. *pneumoniae* distributions describes >96% concordance for all antimicrobials except erythromycin and clindamycin; however, the testing methods used during this time period varied substantially in comparison to the current summary (5). The previously discussed Lovgren et al.’s study, reporting the establishment of the SIREVA EQA, notes MIC concordance of 91.0% by modal MIC and 95.3% by categorical agreement for phase II of the program development (1999–2005) (14). Similarly to the current ICS QC program results, there were a handful of major errors for chloramphenicol and erythromycin. The European Antimicrobial Resistance Surveillance Network (EARS-Net) offers a yearly EQA panel for participating laboratories. The EARS-Net panel focuses on a variety of key antimicrobial-resistant pathogens and recently has included up to one *S*. *pneumoniae* isolate per panel. From 2017–2019, there was one *S*. *pneumoniae* isolate on each yearly panel; very good concordance (>90%) with intended results was reported for most compounds during this time span; however, authors described ongoing issues with concordance for β-lactam-intermediate *S. pneumoniae* isolates (10, 18, 19). Though the number varied depending on the breakpoints applied to our ICS QC results, >20 and >15 errors were identified for penicillin and ceftriaxone/cefotaxime, respectively. The majority of these errors resulted from a difference in one doubling dilution on either side of the breakpoint for susceptible and intermediate, regardless of applied breakpoints.

A limitation of this program, particularly the antimicrobial susceptibility testing portion, is the different testing methods used by each participating laboratory. Though most laboratories use broth microdilution for this exercise, the differing test ranges for each antimicrobial make it increasingly difficult to calculate modal MIC values. Though the concordance for all antimicrobials was high, this underscores the importance of standard protocols to be used across submitting laboratories when calculating values for the final report. Moving forward, many participating laboratories are adopting routine whole-genome sequencing-based identification of serotypes and antimicrobial susceptibilities for *S. pneumoniae*. In this event, the ICS QC program will need to implement minimum quality standards for genomic data to ensure that submitted typing results are of high quality.

The ICS QC program for *S. pneumoniae* has been a successful collaboration for over 20 years, in addition to QC programs for another vaccine-preventable disease such as *Haemophilus influenzae* and *Neisseria meningitidis* (20). Following the success of these programs, an interlaboratory QC program for *emm* typing of Group A Streptococcus (*Streptococcus pyogenes*) began in 2011 (21). More recently, a QC program has been developed for serotyping of Group B Streptococcus (*Streptococcus agalactiae*), beginning with three participating laboratories in 2021 and expanding to five by the 2023 distribution. Ongoing participation in ICS QC programs will ensure the continuation of quality surveillance systems within Arctic populations that experience health disparities.
